# Burden of Food Insecurity and Mental Health Symptoms among Adults with Cardiometabolic Conditions during the COVID-19 Pandemic

**DOI:** 10.3390/ijerph191610077

**Published:** 2022-08-15

**Authors:** Marlene Camacho-Rivera, Jonathan Albury, Karen Chen, Zachary Ye, Jessica Y. Islam

**Affiliations:** 1 Department of Community Health Sciences, School of Public Health, SUNY Downstate Health Sciences University, Brooklyn, NY 11203, USA; 2 CUNY School of Medicine, The City College of New York, New York, NY 10031, USA; 3 College of Medicine, SUNY Downstate Health Sciences University, Brooklyn, NY 11203, USA; 4 H. Lee Moffitt Cancer Center and Research Institute, Tampa, FL 33028, USA

**Keywords:** food insecurity, cardiovascular, diabetes, mental health, COVID-19 pandemic

## Abstract

Our study objectives were to (1) identify the national prevalence and patterns of food insecurity among adults with and without a history of CMCs and (2) determine associations between food insecurity and mental health outcomes among adults with CMCs during the early COVID-19 pandemic period (April–June 2020). We computed prevalence ratios with Poisson regression using the robust estimation of standard errors to identify disparities in the report of food insecurity across demographic groups and by CMC history. Among adults with CMCs, we estimated associations between food insecurity and self-reported mental health symptoms using multinomial logistic regression. Overall, people with CMCs were more likely to be older, White, without employment in the past 7 days, and from the South or an urban environment. We found that the determinants of food insecurity among individuals with cardiometabolic conditions include having: <60 years of age, female sex, Black or Hispanic race/ethnicity, an educational degree lower than a baccalaureate, a household income of <$100,000, and either Medicaid, Indian Health Insurance, or no insurance. Individuals with CMCs and food insecurity also had significantly higher odds of adverse mental health symptoms. The continued clinical screening of food insecurity and mental health, as well as public health interventions, targeted toward individuals with CMCs, should be prioritized as we move through the COVID-19 pandemic.

## 1. Introduction

Food insecurity is defined by the United Nations as lacking “physical and economic access to sufficient safe and nutritious food that meet their food preferences and dietary needs for an active and healthy life” [1]. Increasing severity of food insecurity is associated with a greater incidence of and worse outcomes for cardiometabolic conditions (CMC) [2,3], such as hypertension, stroke, and diabetes [4,5,6]. Food insecurity also affects mental health [7] especially among low-income families [8], promoting feelings of sadness, anxiety, and hopelessness [9], predisposing them to CMCs [10,11]. Poor mental health can lead to lower life expectancy and worse physical health outcomes [12], putting people at increased risk of infections such as COVID-19 [13].

The COVID-19 pandemic exacerbated existing contributors to food insecurity. Public health measures—lockdown, social distancing, and quarantine—restricted physical access to food, especially affecting those already at risk of food insecurity [13]. Many lost their jobs, and without income, people could not afford fresh, nutritious foods, opting instead for non-perishables [14,15]. The pandemic inordinately impacted the essential food supply chain workforce [16]. In the early pandemic, the national food insecurity rate exceeded 20%, compared to 11% pre-pandemic, with rates still elevated to 16.6% as of May 2021 [17].

COVID-19 also disproportionately affects people with existing comorbidities, especially CMCs [18,19,20,21]. According to the CDC, from January to March 2020, over 22% of COVID-19 patients had underlying health conditions, most commonly cardiovascular disease (32%) and diabetes (30%) [19]. Hospitalizations and deaths in these patients were 6 times higher (45.4%) and 12 times higher (19.5%), respectively [19].

Existing studies explore associations between food insecurity during the pandemic and mental health outcomes, using data from surveys of specific hospitals, cities, or states [15,16]. However, few studies examined the relationship between food insecurity and mental health in CMCs on a national level. To address this discrepancy, we used the COVID Impact Survey, a cross-sectional, nationally representative survey evaluating Americans’ physical, economic, and social well-being. This study team previously measured mental health symptoms among patients with chronic health conditions, such as cancer or an immunocompromised system [22,23,24]. Our current objectives are to (1) identify the national prevalence and patterns of food insecurity among adults with and without a history of CMCs and (2) determine the associations between food insecurity and mental health outcomes among adults with CMCs during the early COVID-19 pandemic period (April–June 2020).

## 2. Materials and Methods

### 2.1. COVID-19 Impact Survey

Data for these analyses were obtained from the publicly available COVID-19 Household Impact Survey, conducted by the National Opinion Research Center (NORC) at the University of Chicago. The COVID-19 Household Impact Survey provides national and regional statistics on physical health, mental health, economic security, and social dynamics of a nationally representative sample of the U.S. population [25], identified through the AmeriSpeak^®^ Sample. The survey is designed to provide weekly estimates of the U.S. adult household population nationwide. The COVID-19 Household Impact Survey was collected over three time periods: Week One (20–26 April 2020), Week Two (4–10 May 2020), and Week Three (30 May–8 June 2020). Data from all three time periods were merged for the present analysis.

### 2.2. AmeriSpeak Sample

Funded and operated by NORC at the University of Chicago, AmeriSpeak^®^ is a probability-based panel designed to be representative of the US household population. During the initial recruitment phase of the AmeriSpeak panel, randomly selected US households were sampled using area probability and address-based sampling, with a known, nonzero probability of selection from the NORC National Sample Frame. These sampled households were then contacted by US mail, telephone, and field interviewers (face to face). The panel provides sample coverage of approximately 97% of the US household population. Those excluded from the sample include people with P.O. Box only addresses, some addresses not listed in the US Postal Service Delivery Sequence File, and some newly constructed dwellings. While most AmeriSpeak households participate in surveys via the web, non-internet households were able to participate in AmeriSpeak surveys by telephone. Households without conventional internet access but having web access via smartphones could participate in AmeriSpeak surveys by web. AmeriSpeak panelists participate in NORC studies or studies conducted by NORC on behalf of governmental agencies, academic researchers, and media and commercial organizations. Interviews were conducted in English and Spanish. Interviews were conducted with adults age 18 and over representing the 50 states and the District of Columbia. Panel members were randomly drawn from AmeriSpeak. In households with more than one adult panel member, only one was selected at random for the sample. Invited panel members were given the option to complete the survey online or by telephone with a NORC telephone interviewer. The number of participants invited, and the percentage of interviews completed by week are as follows: 11,133 invited with 19.7% interviews completed during Week 1; 8570 invited with 26.1% interviews completed (Week 2); and 10,373 invited with 19.7% interviews completed (Week 3). The analytic sample included 10,760 adults nationwide. The final analytic sample was weighted to reflect the US population of adults aged 18 years and over. The demographic weighting variables were obtained from the 2020 Current Population Survey.

### 2.3. Cardiometabolic Conditions

We categorized adults as having a cardiometabolic condition based on self-report. Participants were asked the following question: “Has a doctor or other health care provider ever told you that you have any of the following: Diabetes; High blood pressure or hypertension; Heart disease, heart attack or stroke; Asthma; Chronic lung disease or COPD; Bronchitis or emphysema; Allergies; a Mental health condition; Cystic fibrosis; Liver disease or end-stage liver disease; Cancer; a Compromised immune system; or Overweight or obesity.” We defined those who selected diabetes, high blood pressure or hypertension, heart disease/heart attack/stroke, and liver disease or end-stage liver disease as an adult with a cardiometabolic condition.

### 2.4. Food Insecurity

Our primary outcome for this analysis was a self-report of food insecurity. First, we defined food insecurity using the following questionnaire item; Please indicate whether the following statements were often true, sometimes true, or never true for you or your household over the past 30 days: (1) We worried our food would run out before we got money to buy more, (2) The food that we bought just didn’t last and we didn’t have money to get more. Respondents who indicated either of those statements were often true or sometimes true were categorized as food insecure. Additionally, respondents were categorized as food insecure if they indicated they received or applied for income assistance from a food pantry or the Supplemental Nutrition Assistance Program (SNAP) in the past seven days.

### 2.5. Mental Health Symptoms

The mental health outcome for this analysis was a self-report of mental health symptoms in the past 7 days. Participants were categorized based on their responses to the following questionnaire item; In the past 7 days, how often have you: (A) felt nervous, anxious, or on edge, (B) felt depressed, (C) felt lonely, (D) felt hopeless about the future, (E) had physical reactions such as sweating, trouble breathing, nausea or a pounding heart when thinking about your experience with the coronavirus pandemic. For each of the statements, the response options were: (1) not at all or less than 1 day, (2) 1–2 days, (3) 3–4 days, and (4) 5–7 days.

### 2.6. Covariates

The following covariates were included in the multivariable analyses: age (18–29, 30–44, 25–59, 60+), gender (male/female), marital status (married/living with a partner, widowed/divorced/separated, never married), education categories (no high school diploma, HS graduate or equivalent, some college, baccalaureate degree or above), employment status (employed/unemployed) household income (<$50,000, $50,000–$100,000, ≥$100,000), population density (rural, suburban, urban), census region (Northeast, Midwest, South, West), and insurance type (purchased plan/employer-sponsored/TRICARE/Medicaid/Medicare/Dually-eligible/VA/uninsured).

### 2.7. Data Analysis

Descriptive statistics are summarized, by cardiometabolic condition status, in percentages among all respondents and include a margin of error of +/− 3.0 percentage points at the 95% confidence intervals. We used chi-square tests to compare the prevalence of food insecurity among adults with cardiometabolic conditions compared to the general U.S. adult population by key demographic covariates. To identify demographic groups that may be more likely to report food insecurity, we estimated the determinants of food insecurity among adults with cardiometabolic conditions. We computed prevalence ratios with Poisson regression using robust estimation of standard errors [26,27,28]. Potential variables for inclusion in the model were assessed using available sociodemographic variables and bivariate Poisson regression analysis. Due to the exploratory nature of this analysis using a predictive framework, an arbitrary *p*-value of <0.10 was used as criteria to include the variable in the multivariable Poisson regression model. For multivariable Poisson regression models, adjusted prevalence ratios (aPR) and 95% confidence intervals (CIs) for each independent variable were calculated.

Next, we used multinomial logistic regression to evaluate the associations between food insecurity and mental health symptoms reported in the last 7 days among adults with cardiometabolic conditions. We adjusted for age, sex, race/ethnicity, annual household income, education, insurance status, employment status, and area of residence (urban/rural). To address concerns regarding existing mental health symptoms before the COVID-19 pandemic, we conducted a sensitivity analysis to evaluate mental health symptoms among those without a history of a mental health condition based on self-report. We were able to assess the history of a mental health condition through the following question: “Have you ever been diagnosed by a doctor or health care provider ever said you have a mental health condition?” Although “mental health condition” may include several conditions, based on participant responses to this question, we excluded participants with a history of clinical depression and anxiety using this approach (*n* = 713). Based on the exploratory nature of this analysis, we did not include an adjustment for multiple comparisons [29,30]. All statistical analyses were conducted using Stata IC 15 (StataCorp LLC, College Station, TX, USA). Sampling weights were applied to provide results that were nationally representative of the U.S. adult population.

## 3. Results

A total of 10,760 adults were included in the final study population, 38.7% of whom had reported having a cardiometabolic condition (Table 1).

Overall, people with cardiometabolic conditions were more likely to be older, White, without employment in the past 7 days, from the South, or in an urban environment. A majority of adults with cardiometabolic conditions were either on employer-sponsored insurance or Medicare and had a household income of less than $30,000. Responses to the measures of food insecurity are also described in Table 1. Among adults with a cardiometabolic condition, 26.1% worried about running out of food before having money to buy more, 21.4% reported that the food they bought did not last and they did not have money to get more, 14.7% either received or applied for Supplemental Nutrition Assistance Program, and 9.3% either received or applied for assistance from a food pantry. Among adults without cardiometabolic conditions, 28.6% worried about running out of food before having money to buy more, 21.5% reported that the food they bought did not last and they did not have money to get more, 12.1% either received or applied for Supplemental Nutrition Assistance Program, and 6.7% either received or applied for assistance from a food pantry.

Compared to adults without a cardiometabolic condition, the prevalence of food insecurity was higher for adults with a cardiometabolic condition within all age groups (67.3%, 52.5%, 35.5%, and 24.0% of adults aged 18–29, 30–44, 45–59, and over 60, respectively). Females (41.7%), never married individuals (52.3%), individuals with a household income of $30,000–$50,000, and individuals with either a Purchased Plan (23.3%), Medicare (31.6%), or Indian Health Service (90.3%) insurance types had a significantly higher prevalence of food insecurity among those with a cardiometabolic condition versus those without. Males with cardiometabolic conditions had a lower prevalence of food insecurity compared to males without a cardiometabolic condition. There were no significant differences found within subgroups of race/ethnicity, employment status in the last 7 days, education, region, or population density (Table 2).

As shown in Table 3, in our analysis of determinants of food insecurity among adults with cardiometabolic conditions, we found the following groups to have a significantly higher adjusted prevalence of food insecurity: age 18–29, age 30–44, and age 45–49 compared to those age 60 and above; female compared to males; non-Hispanic Black and Hispanic race/ethnicity compared to White race/ethnicity. Individuals with Medicaid, Indian Health Service, and no insurance had a higher prevalence of food insecurity compared to those with VA insurance. Individuals without a high school diploma, high school graduate, and some college had a higher prevalence of food insecurity compared to those with a baccalaureate or above; individuals with a household income <$30,000, $30,000–$50,000, $50,000–$75,000, and $75,000–$100,000 had a higher prevalence of food insecurity compared to individuals with a household income of >$100,000. On the other hand, groups with cardiometabolic conditions that had a significantly lower adjusted prevalence of food insecurity included those with employer-sponsored insurance, Medicare insurance, and employment status of employed/self-employed.

**Figure 1:**
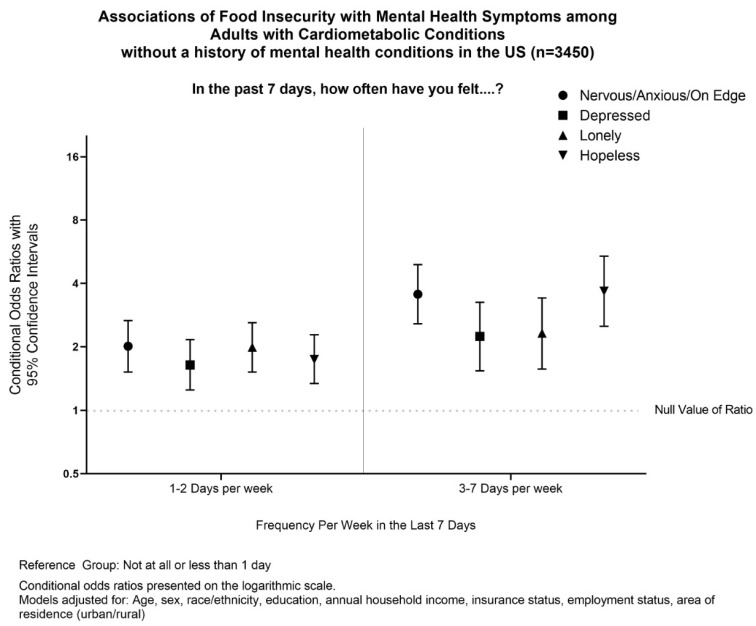
Associations of Food Insecurity with Mental Health symptoms among Adults with Cardiometabolic Conditions without a history of mental health in the US (*n* = 3450).

Individuals with cardiometabolic conditions and food insecurity also had significantly higher odds of mental health symptoms in the last 7 days than those without food insecurity. For all patients with cardiometabolic diseases, the odds of feeling nervous/anxious/on edge, depressed, lonely, or hopeless for 3–7 days per week were 3.65, 3.02, 3.05, and 4.94, respectively. In patients without existing mental health conditions, the odds of feeling nervous/anxious/on edge, depressed, lonely, or hopeless for 3–7 days per week were 3.55, 2.24, 2.32, and 3.67, respectively (Figure 1).

## 4. Discussion

Prior to the pandemic, an estimated 10.5% of US households were food insecure at least sometime during the year 2019; food insecurity was most prevalent among Black and Hispanic populations as well as low-income households [31]. Among survey respondents, the self-report of food insecurity ranged from 9.7% to 27.4%, indicating an increase in self-reported food insecurity during the early pandemic period. Access to food was further limited during the pandemic due to an increase in demand for food, disruptions in the food supply, worries about the risk of infection from leaving the home and shopping in the grocery store, as well as the fear of viral contamination of food. Food aid services also had fewer volunteers due to quarantine orders [15]. These factors exacerbated existing economic and health disparities that contribute to food insecurity.

We found that the determinants of food insecurity among individuals with cardiometabolic conditions include having: <60 years of age, female sex, Black or Hispanic race/ethnicity, an educational degree lower than a baccalaureate, a household income of <$100,000, and either Medicaid, Indian Health Insurance, or no insurance. A previous study reporting social determinants of food insecurity among adults with atherosclerotic cardiovascular disease showed similar high-risk characteristics. However, in contrast, our results showed a lower adjusted prevalence of food insecurity among individuals with employer-sponsored insurance and no significant difference in the adjusted prevalence of food insecurity within marital status [32].

Since the start of the COVID-19 pandemic, there has been an increased prevalence of depression, anxiety, distress, and insomnia globally. These findings have been most apparent in patients with chronic disease, individuals undergoing quarantine, patients suspected of COVID-19, and healthcare workers [33]. Our study revealed that among individuals with cardiometabolic conditions without an existing mental health condition, the odds of having mental health symptoms were greater in those experiencing food insecurity compared to those without food insecurity. Many studies have shown that food insecurity significantly increases the risk of depressive symptoms and stress, likely due to concerns of not having enough food and feeling deprived. Specifically, in North America, food insecurity is also associated with increased anxiety [34]. Food insecurity may be related to atherosclerotic cardiovascular risk disease through a psychological/mental health pathway in which food insecurity leads to increased anxiety and depression, thus increasing the risk of cardiovascular disease [35]. During the COVID-19 pandemic, the rise in food insecurity, as well as lifestyle changes, worries of unemployment, and economic instability as a result of the COVID-19 pandemic, have also contributed to the rise in mental health symptoms. A study examining associations between food worry and mental health during the pandemic in Canada revealed that food worry was more prevalent in households experiencing financial worry, and that food worry may be associated with mental health through such financial concerns. However, even after adjusting for financial worry, food worry still had a strong association with higher odds of suicidal feelings [36]. The significantly higher odds of having mental health symptoms as seen in our data may be a result of the compounded effects that food insecurity, financial worries, and non-financial worries have on the mental health of patients with cardiometabolic conditions during the pandemic.

To the best of our knowledge, this is the first study to identify the prevalence of food insecurity among adults with and without cardiometabolic conditions as well as determine associations between food insecurity and mental health symptoms among individuals with cardiometabolic conditions during the early COVID-19 pandemic. Our study utilized data from the COVID-19 Household Impact Survey, which is a large, nationally representative sample that allows for generalization to the U.S. population. The data also provided opportunities to analyze and adjust for multiple sociodemographic variables, allowing us to identify risk factors for food insecurity among individuals with cardiometabolic conditions. Given the associations between food insecurity, cardiometabolic conditions, and mental health during the COVID-19 pandemic, our study suggests a need for interventions, improved access, and linkage of care for the most vulnerable subgroups. Screening for food insecurity at health visits allows healthcare providers to refer patients to appropriate social services such as the Supplemental Nutrition Assistance Program and food pantries. Such programs can also be expanded for patients with qualified cardiometabolic conditions. Mental health screenings are crucial in identifying patients who may need counseling or psychiatric services.

Despite the study’s strengths, the results should be interpreted in the context of its limitations. While the sampling design of the COVID-19 Household Impact Survey is designed to cover 97% of households in the US and provide nationally representative survey estimates of US adults, it is possible that individuals in households without PO boxes, individuals in households without access to internet or smartphones, or individuals who are unstably housed were more likely to experience food insecurity, which would provide an underestimate of the burden of food insecurity during the COVID-19 pandemic. Further, data collected from the COVID-19 Household Impact Survey were self-reported, which can be affected by biases that lead to inaccurate reporting of responses. Due to the cross-sectional study design of the survey, we were unable to establish causal relationships or longitudinal changes between food insecurity, cardiometabolic conditions, and mental health symptoms. Additionally, due to the time period of the COVID-19 Household Impact Survey early in the pandemic, and without measurements of food insecurity prior to the COVID-19 pandemic, we cannot define how food insecurity has changed through the pandemic period. Further studies are needed to investigate long-term changes with ongoing assessments of food insecurity, sociodemographic factors, cardiometabolic conditions, and mental health outcomes.

## 5. Conclusions

We observed that during the COVID-19 pandemic, adults with CMCs were more likely to report food insecurity, and, consequently, associated mental health symptoms compared to individuals without a history of cardiometabolic conditions. These findings have important implications given the associations between food insecurity, mental health symptoms, and poor chronic disease self-management before the COVID-19 pandemic. Longitudinal studies are necessary to determine how to best examine the persistence of food insecurity and mental health symptoms throughout and beyond the COVID-19 pandemic, as well as their long-term implications for CMC management and health consequences (e.g., emergency department use, hospitalizations, and quality of life). Screening for food insecurity at health visits allows healthcare providers to refer patients to appropriate social services such as the Supplemental Nutrition Assistance Program and food pantries; special attention should be paid to expanding these services to individuals living with cardiometabolic conditions. Mental health screenings and policies for expanding access to mental health services are crucial in identifying individuals who may need counseling or psychiatric services due to a history of mental health conditions, the mental health consequences of the management of CMCs, adverse social determinants of health due to the COVID-19 pandemic, or a combination of all of the above.

## Figures and Tables

**Table 1:** Characteristics of COVID Impact Survey respondents (*n* = 10,760), a nationally representative survey of the US, stratified by cardiometabolic disease status (April–June 2020).

	Total		Adults with Cardiometabolic * Conditions (*n* = 4163)		Adults without Cardiometabolic Conditions (*n* = 6597)	
	Col %	95% CI	Col %	95% CI	Col %	95% CI
**Age**						
18–29	20.5	19.3, 21.8	7	5.8, 8.4	28.7	27.0, 30.6
30–44	25.4	24.4, 26.5	14.8	13.5, 16.3	31.9	30.4, 33.4
45–59	24.3	23.2, 25.4	27.2	25.4, 29.0	22.5	21.2, 23.9
60+	29.8	28.6, 30.9	51	49.0, 53.1	16.9	15.7, 18.0
**Sex**						
Male	48.3	47.0, 49.6	51.8	49.8, 53.9	46.2	44.4, 47.9
Female	51.7	50.4, 53.0	48.2	46.1, 50.2	53.8	52.1, 55.6
**Race/Ethnicity**						
White, NH	62.3	61.0, 63.6	64.3	62.2, 66.3	61.2	59.4, 62.9
Black, NH	11.9	11.1, 12.8	15.2	13.7, 16.8	9.9	8.9, 10.9
Hispanic	16.7	15.6, 17.8	12.4	10.9, 14.0	19.3	17.8, 20.8
Other, NH	8.6	7.8, 9.4	7.5	6.4, 8.7	9.3	8.2, 10.4
**Employed in the past 7 days**	49.7	48.4, 51.1	36.7	34.8, 38.7	57.6	55.9, 59.3
**Education**						
No HS Diploma	9.8	8.8, 10.8	10.1	8.6, 11.8	9.6	8.3, 11.0
HS Graduate	28.2	27.0, 29.6	30.9	29.0, 32.9	26.6	25.0, 28.4
Some College	27.7	26.7, 28.7	29.7	28.1, 31.3	26.5	25.3, 27.8
Baccalaureate or above	34.3	33.1, 35.5	29.3	27.5, 31.2	37.3	35.7, 38.9
**Household Income**						
<$30,000	26.8	25.5, 28.0	31	29.1, 32.9	24.2	22.7, 25.8
$30,000–$50,000	19	18.1, 20.0	20.9	19.3, 22.6	17.9	16.7, 19.2
$50,000–$75,000	18.5	17.5, 19.5	16.5	15.0, 18.0	19.7	18.4, 21.0
$75,000–$100,000	13.6	12.7, 14.5	11.8	10.6, 13.2	14.7	13.5, 15.9
≥$100,000	22.1	21.1, 23.2	19.8	18.2, 21.5	23.5	22.1, 25.0
**Region**						
Northeast	17.4	16.4, 18.5	16.9	15.3, 18.6	17.8	16.4, 19.2
Midwest	20.7	19.8, 21.7	22	20.5, 23.7	20	18.8, 21.2
South	38	36.7, 39.3	40.7	38.7, 42.7	36.4	34.7, 38.1
West	23.8	22.8, 24.9	20.4	18.8, 22.1	25.9	24.5, 27.4
**Population Density**						
Rural	9.1	8.4, 9.8	11.6	10.3, 12.9	7.6	6.8, 8.5
Suburban	18.8	17.8, 19.7	20.5	18.9, 22.1	17.7	16.6, 19.0
Urban	72.2	71.0, 73.3	68	66.1, 69.8	74.7	73.3, 76.1
**Insurance Type or Health Coverage Plans**						
Purchased Plan	17.4	16.4, 18.5	20.9	19.2, 22.7	15.3	14.1, 16.7
Employer-Sponsored	51.7	50.3, 53.0	43.4	41.4, 45.5	56.7	54.9, 58.4
TRICARE	4.9	4.4, 5.4	6.1	5.2, 7.0	4.2	3.7, 4.9
Medicaid	23.5	22.4, 24.7	30.1	28.2, 32.1	19.6	18.2, 21.0
Medicare	25.3	24.2, 26.4	44.5	42.5, 46.6	13.6	12.5, 14.7
Dually Eligible (Medicare and Medicaid)	9.7	9.0, 10.4	17.8	16.3, 19.5	4.8	4.2, 5.5
VA	4.5	4.0, 5.0	6.8	5.9, 7.9	3.1	2.6, 3.6
Indian Health Service	1.2	0.9, 1.6	2	1.4, 2.9	0.7	0.5, 1.1
No insurance	8.8	8.1, 9.6	5.6	4.7, 6.7	10.8	9.7, 11.9
**Food Insecurity Measures**						
**Over the past 30 days, we worried our food would run out before we got money to buy more**						
Often true	6.2	5.5, 6.9	5.1	4.2, 6.1	6.8	6.0, 7.7
Sometimes true	20.3	19.1, 21.4	21	19.3, 22.8	19.8	18.4, 21.4
Never true	73.6	72.3, 74.8	73.9	72.0, 75.8	73.4	71.7, 74.9
**Over the past 30 days, the food that we bought just didn’t last, and we didn’t have money to get more**						
Often true	4.5	4.0, 5.1	4.3	3.5, 5.2	4.7	4.0, 5.5
Sometimes true	16.9	15.9, 18.0	17.1	15.4, 18.9	16.8	15.5, 18.2
Never true	78.6	77.4, 79.7	78.7	76.8, 80.4	78.5	77.0, 80.0
**In the past 7 days, have you received/applied for any of the following forms of income assistance, or not?: SNAP (Supplemental Nutrition Assistance Program)**						
Received	10.7	10.0, 11.5	12.1	10.9, 13.5	9.9	9.0, 10.9
Applied for	2.2	1.8, 2.8	2.6	1.8, 3.8	2	1.5, 2.6
Tried to apply for	2	1.6, 2.4	1.5	1.1, 2.2	2.2	1.8, 2.7
Did not receive nor apply for any	85.1	84.1, 86.0	83.7	82.1, 85.3	85.9	84.8, 87.0
**In the past 7 days, have you received/applied for any of the following forms of income assistance, or not?: A food pantry**						
Received	6.8	6.1, 7.4	8.4	7.4, 9.6	5.8	5.0, 6.6
Applied for	0.9	0.7, 1.2	0.9	0.6, 1.4	0.9	0.6, 1.4
Tried to apply for	1	0.8, 1.4	1.6	1.1, 2.3	0.7	0.5, 1.0
Did not receive nor apply for any	91.3	90.5, 92.0	89.1	87.7, 90.3	92.6	91.7, 93.5

* Cardiometabolic conditions include diabetes, high blood pressure, heart disease, and end-stage kidney disease; NH is non-Hispanic, HS is high school, VA is Veterans Administration.

**Table 2:** Prevalence of food insecurity by cardiometabolic condition status among COVID Impact Survey respondents (*n* = 10,760), a nationally representative survey of the US (April–June 2020).

	Total		Adults with Cardiometabolic Conditions		Adults without Cardiometabolic Conditions		
	Row %	95% CI	Row %	95% CI	Row %	95% CI	*p*
**Age**							
18–29	47.3	43.7, 51.0	67.3	58.7, 74.8	44.4	40.5, 48.3	<0.001
30–44	42.4	40.1, 44.8	52.5	47.6, 57.4	39.6	36.9, 42.2	<0.001
45–59	28.9	26.6, 31.2	35.5	31.9, 39.3	24	21.3, 27.0	<0.001
60+	21.3	19.5, 23.2	24.0	21.6, 26.6	16.2	13.8, 19.0	<0.001
**Sex**							
Male	30	28.1, 31.9	27.6	25.0, 30.3	31.6	29.1, 34.3	0.032
Female	37.4	35.7, 39.2	41.7	38.8, 44.7	35.1	33.0, 37.3	<0.001
**Marital Status**							
Married/Living with Partner	28.2	26.6, 29.8	27.3	24.8, 29.9	28.7	26.7, 30.8	0.40
Widowed/Divorced/Separated	36.4	33.7, 39.2	38.1	34.4, 41.9	34.2	30.3, 38.2	0.16
Never Married	45.2	42.2, 48.2	52.3	47.1, 57.5	42.8	39.3, 46.4	0.003
**Race/Ethnicity**							
White, NH	26.1	24.6, 27.6	27.3	25.1, 29.6	25.3	23.4, 27.3	0.19
Black, NH	51.4	47.6, 55.1	49.9	44.3, 55.4	52.8	47.6, 57.9	0.45
Hispanic	49.7	46.0, 53.5	50.3	43.4, 57.1	49.5	45.1, 54.0	0.86
Asian, NH	33.7	26.8, 41.5	38.6	26.5, 52.3	32.1	23.9, 41.5	0.42
Other	37.1	31.6, 42.8	35.9	28.1, 44.6	37.9	30.7, 45.7	0.73
**Employment Status in the Past 7 Days**							
Unemployed	40.0	38.2, 41.9	39.7	37.1, 42.3	40.3	37.7, 42.9	0.74
Employed	27.8	26.0, 29.6	25.4	22.5, 28.5	28.7	26.5, 31.0	0.09
**Education**							
No HS Diploma	65.0	59.6, 69.9	64.4	56.2, 71.8	65.3	58.2, 71.8	0.85
HS Graduate	43.5	40.7, 46.4	42.9	39.0, 46.9	43.9	40.0, 47.9	0.72
Some College	35.0	33.2, 36.9	32.8	30.1, 35.6	36.5	34.2, 39.0	0.05
Baccalaureate or above	16.0	14.5, 17.6	16.6	14.2, 19.4	15.7	13.9, 17.7	0.58
**Household Income**							
<$30,000	63.9	61.2, 66.5	63.5	59.8, 67.0	64.2	60.4, 67.9	0.78
$30,000–$50,000	36.2	33.5, 39.0	31.8	27.9, 35.9	39.4	35.8, 43.2	0.01
$50,000–$75,000	27.5	24.7, 30.4	25.7	21.2, 30.9	28.3	25.0, 31.9	0.4
$75,000–$100,000	18.4	15.6, 21.6	18.0	13.4, 23.6	18.6	15.2, 22.7	0.83
≥$100,000	10.2	8.5, 12.1	8.6	6.6, 11.1	11	8.7, 13.7	0.16
**Region**							
Northeast	32.6	29.4, 36.0	34.9	29.8, 40.3	31.3	27.2, 35.7	0.29
Midwest	31.5	29.2, 33.9	34.2	30.5, 38.1	29.7	26.8, 32.8	0.07
South	36.6	34.4, 38.8	35.2	32.1, 38.5	37.5	34.6, 40.5	0.31
West	32.4	29.8, 35.0	32.4	28.2, 36.9	32.3	29.2, 35.6	0.98
**Population Density**							
Rural	37.2	33.2, 41.3	41.3	35.5, 47.4	33.3	28.2, 38.9	0.05
Suburban	32.6	30.0, 35.3	32.0	28.1, 36.1	33	29.6, 36.7	0.7
Urban	33.7	32.2, 35.3	33.9	31.5, 36.4	33.6	31.7, 35.7	0.87
**Insurance Type or Health Coverage Plans**							
Purchased Plan	26.7	23.9, 29.8	23.3	19.5, 27.5	29.5	25.5, 34.0	0.04
Employer-Sponsored	21.1	19.5, 22.7	20.5	17.9, 23.3	21.3	19.4, 23.4	0.64
TRICARE	27.2	22.4, 32.6	25	18.2, 33.1	29.2	22.7, 36.6	0.43
Medicaid	67.1	64.5, 69.6	66.5	62.9, 70.0	67.6	63.8, 71.2	0.69
Medicare	29.7	27.5, 32.0	31.6	28.9, 34.5	25.9	22.4, 29.8	0.02
Dually Eligible (Medicare and Medicaid)	56.8	52.8, 60.8	59.3	54.4, 64.1	51.2	44.2, 58.1	0.06
VA	31.7	26.3, 37.6	32.3	24.9, 40.7	30.8	23.6, 39.0	0.79
Indian Health Service	83.0	74.1, 89.3	90.3	80.9, 95.4	70.7	51.4, 84.6	0.02
No insurance	56.5	51.9, 61.1	55	46.0, 63.6	57	51.6, 62.3	0.7

Cardiometabolic conditions include diabetes, high blood pressure, heart disease, and end-stage kidney disease; NH is non-Hispanic, HS is high school, and VA is Veterans Administration.

**Table 3:** Determinants of food insecurity among adults with cardiometabolic conditions in the COVID Impact Survey, a nationally representative survey of the US (*n* = 4163) (April–June 2020).

	Unadjusted PR	95% CI	Adjusted PR	95% CI
**Age**				
18–29	2.80	2.39–3.29	1.95	1.61–2.36
30–44	2.19	1.90–2.52	1.99	1.72–2.32
45–49	1.48	1.27–1.71	1.38	1.20–1.59
60+	Ref.		Ref.	
**Sex**				
Male	Ref.		Ref.	
Female	1.52	1.34–1.70	1.16	1.05–1.28
**Marital Status**				
Married/Living with Partner	Ref.		Ref.	
Widowed/Divorced/Separated	1.40	1.22–1.60	1.09	0.97–1.24
Never Married	1.92	1.67–2.20	1.13	0.99–1.27
**Race/Ethnicity**				
White, NH	Ref.		Ref.	
Black, NH	1.83	1.59–2.10	1.21	1.05–1.40
Hispanic	1.84	1.57–2.16	1.26	1.10–1.44
Asian, NH	1.41	0.99–2.01	1.43	1.00–2.03
Other, NH	1.32	1.03–1.69	0.95	0.80–1.12
				0.69–1.12
Insurance Type *				
Purchased Plan	0.62	0.51–0.74	0.94	0.79–1.12
Employer-Sponsored	0.45	0.39–0.52	0.83	0.70–0.98
TRICARE	0.70	0.52–0.95	0.80	0.64–1.00
Medicaid	3.10	2.77–3.48	1.77	1.48–2.12
Medicare	0.87	0.77–0.98	0.76	0.59–0.99
Dually Eligible (Medicare and Medicaid) †	1.99	1.79–2.23	1.42	1.9–1.85
VA	0.93	0.72–1.20	-	
Indian Health Service	2.69	2.44–2.96	1.41	1.18–1.69
No insurance	1.66	1.39–1.97	1.37	1.10–1.69
**Employment Status**				
Not Employed	Ref.		Ref.	
Employed/Self-Employed	0.64	0.55–0.73	0.77	0.68–0.87
**Education**				
No HS Diploma	3.87	3.17–4.72	1.51	1.23–1.86
HS Graduate	2.58	2.15–3.09	1.40	1.18–1.67
Some College	1.97	1.65–2.35	1.32	1.12–1.56
Baccalaureate or above	Ref.		Ref.	
**Household Income**				
<$30,000	7.40	5.68–9.64	3.28	2.46–4.37
$30,000–<$50,000	3.70	2.78–4.94	2.47	1.85–3.29
$50,000–<$75,000	2.99	2.18–4.13	2.40	1.77–3.25
$75,000–<$100,000	2.09	1.43–3.07	1.95	1.39–2.74
≥$100,000	Ref.		Ref.	
**Region**				
Northeast	Ref.		-	
Midwest	0.98	0.81–1.18		
South	1.01	0.85–1.21		
West	0.93	0.76–1.14		
**Population Density**				
Rural	1.22	1.04–1.43	1.10	0.97–1.28
Suburban	0.94	0.81–1.09	1.03	0.91–1.17
Urban	Ref.		Ref.	

* Insurance variables modeled as binary; † Not included in the fully adjusted model due to collinearity with Medicaid; NH is non-Hispanic, HS is high school, and VA is Veterans Administration.

## Data Availability

Publicly available datasets were analyzed in this study. The data can be found here: https://www.covid-impact.org/results (accessed on 14 April 2022).
